# How frequent is osteogenesis imperfecta in patients with idiopathic osteoporosis?: Case reports: Erratum

**DOI:** 10.1097/MD.0000000000008385

**Published:** 2017-10-20

In the article, “How frequent is osteogenesis imperfecta in patients with idiopathic osteoporosis?: Case reports”,^[1]^ which appeared in Volume 96, Issue 35 of *Medicine*, the authors would like to provide an updated Table 1 with information essential to the article. The new version is included below.

**Table 1:**
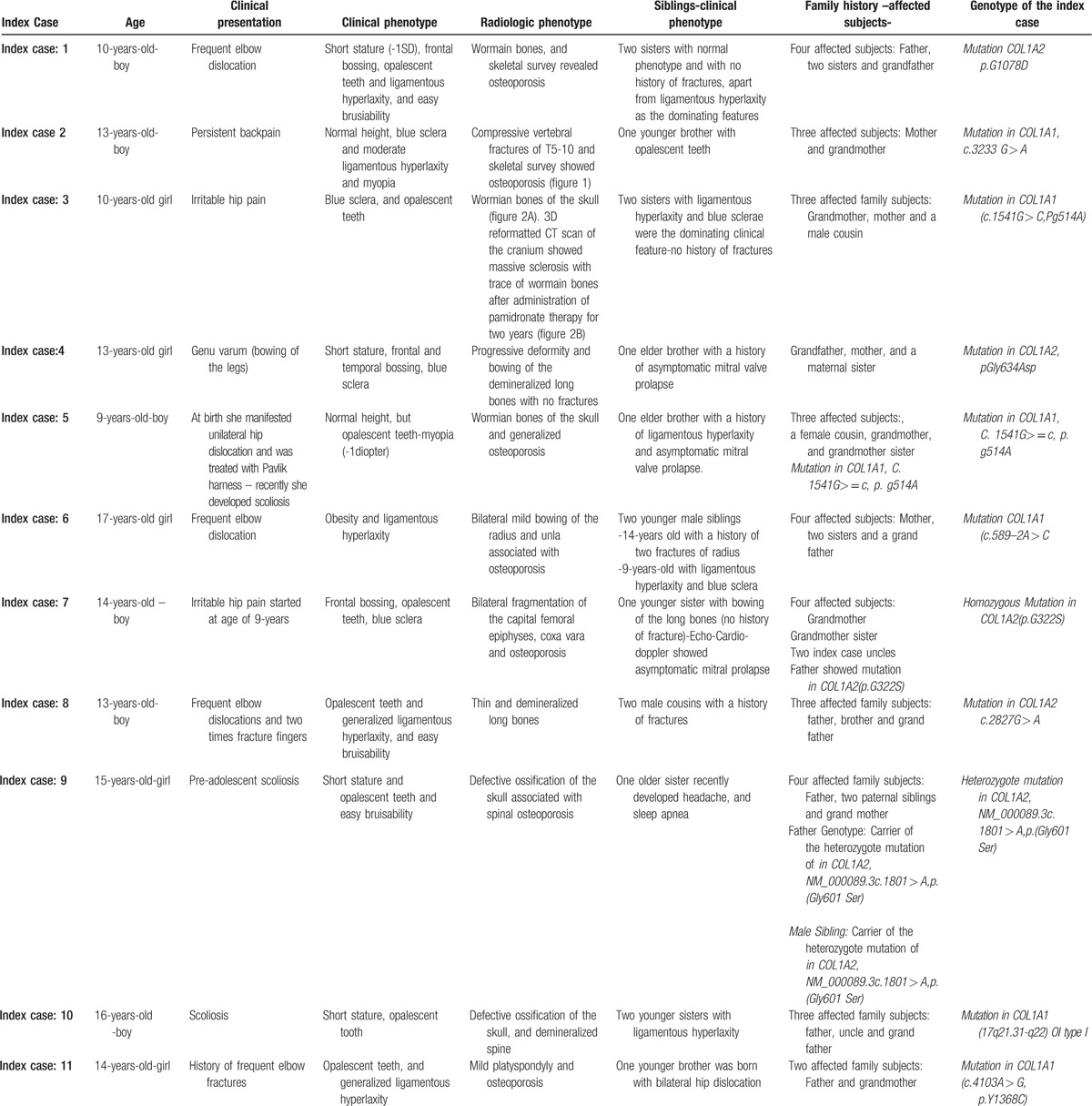
Describes the clinical and radiographic phenotype and the genotype of the index cases-and siblings.
